# Transcriptome‐wide association identifies *KLC1* as a regulator of mitophagy in non‐syndromic cleft lip with or without palate

**DOI:** 10.1002/imt2.262

**Published:** 2024-12-20

**Authors:** Shu Lou, Guirong Zhu, Changyue Xing, Shushu Hao, Junyan Lin, Jiayi Xu, Dandan Li, Yifei Du, Congbo Mi, Lian Sun, Lin Wang, Meilin Wang, Mulong Du, Yongchu Pan

**Affiliations:** ^1^ State Key Laboratory of Cultivation Base of Research, Prevention and Treatment for Oral Diseases Nanjing Medical University Nanjing China; ^2^ Department of Orthodontics, Affiliated Hospital of Stomatology Nanjing Medical University Nanjing China; ^3^ Jiangsu Province Engineering Research Center of Stomatological Translational Medicine Nanjing Medical University Nanjing China; ^4^ The First Affiliated Hospital of Xinjiang Medical University Wulumuqi China; ^5^ State Key Laboratory of Reproductive Medicine Nanjing Medical University Nanjing China; ^6^ Department of Genetic Toxicology, the Key Laboratory of Modern Toxicology of Ministry of Education, Center for Global Health, School of Public Health Nanjing Medical University Nanjing China; ^7^ Department of Biostatistics, Center for Global Health, School of Public Health Nanjing Medical University Nanjing China

## Abstract

This study investigated pathogenic genes associated with non‐syndromic cleft lip with or without cleft palate (NSCL/P) through transcriptome‐wide association studies (TWAS). By integrating expression quantitative trait loci (eQTL) data with genome‐wide association study (GWAS) data, we identified key susceptibility genes, including *KLC1*. Notably, the variant rs12884809 G>A was associated with an increased risk of NSCL/P by enhancing the binding of the transcription factor ELK1 to the *KLC1* promoter, thereby activating its expression. This alteration in *KLC1* expression subsequently impacted mitophagy, leading to significant changes in cellular behavior and zebrafish morphology. Our findings illuminate the genetic mechanisms underlying NSCL/P and provide valuable insights for future prevention strategies and a deeper understanding of this condition.

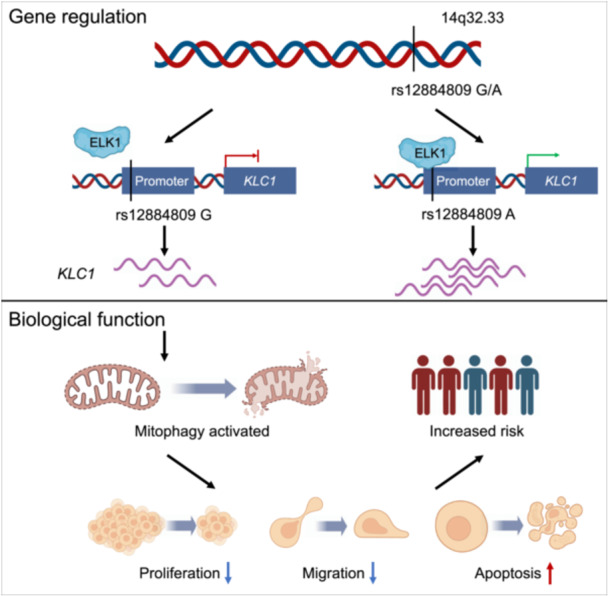

To the Editor,

Non‐syndromic cleft lip with or without palate (NSCL/P) is one of the most prevalent congenital craniofacial anomalies, affecting approximately one in 700 live births worldwide. This condition arises from the incomplete fusion of symmetric facial processes during a complex sequence of cellular growth and migration and is driven by a multifactorial etiology that includes both genetic and environmental factors [1].

To uncover the genetic underpinnings of NSCL/P, genome‐wide association studies (GWAS) have been extensively employed, leading to the identification of susceptibility loci and novel biological mechanisms. For instance, our previous GWAS identified 16p13.3 as a new risk locus [2]. Despite these achievements, significant challenges persist, particularly in enhancing the statistical power and biological interpretation of GWAS results [3]. Furthermore, the complex architecture of linkage disequilibrium (LD) complicates the reliable identification of causal variants and their relationships with disease [4]. Therefore, it is crucial to integrate additional approaches with GWAS to gain a more comprehensive understanding of the biological underpinnings of disease risk.

In light of these challenges, transcriptome‐wide association studies (TWAS) emerge as a promising tool. By linking expression quantitative trait loci (eQTL) with GWAS summary statistics, TWAS can help identify likely target genes and novel loci that might be missed by GWAS alone [5]. For example, Benjamin et al. utilized TWAS and other multi‐omic analyses to pinpoint genes associated with schizophrenia risk [6]. A previous TWAS of NSCL/P identified nine genes implicated in a glutathione synthesis and drug detoxification pathway [7]. Nevertheless, research applying TWAS to NSCL/P has been relatively limited.

To address these gaps, the present study systematically applies the TWAS method to prioritize potential pathogenic genes involved in NSCL/P. Furthermore, functional experiments were conducted to elucidate the underlying mechanisms, providing novel evidence for the genetic basis of NSCL/P.

## RESULTS AND DISCUSSION

### GWAS and TWAS reveal novel genes associated with NSCL/P

We conducted a two‐stage GWAS of NSCL/P, including 1,069 cases and 1,724 controls with 3,620,909 common variants. The quantile–quantile plots indicated minimal inflation (λ_StageI_ = 1.028, λ_StageII_ = 1.020, λ_Meta_ = 1.043, Figure S1A−C). Meta‐analysis revealed significant associations with NSCL/P at 1q32.2 (index by rs72741048, *p* = 9.99 × 10^−11^), 2p24.2 (index by rs6758077, *p* = 1.85 × 10^−^^10^), and 17p13.1 (index by rs9900753, *p* = 3.71 × 10^−8^, Figure S1D−F). Marginal significance was observed at 19q13.11 (index by rs1345417: *p* = 1.42 × 10^−^^7^) and 20q12.1 (index by rs12651896: *p* = 1.81 × 10^−^^7^, Figure S1D−F). The SNP‐based heritability (h^2^_SNP_) of NSCL/P was 0.391 (SE = 0.1256), as consistent with the previous study [8].

**FIGURE 1:**
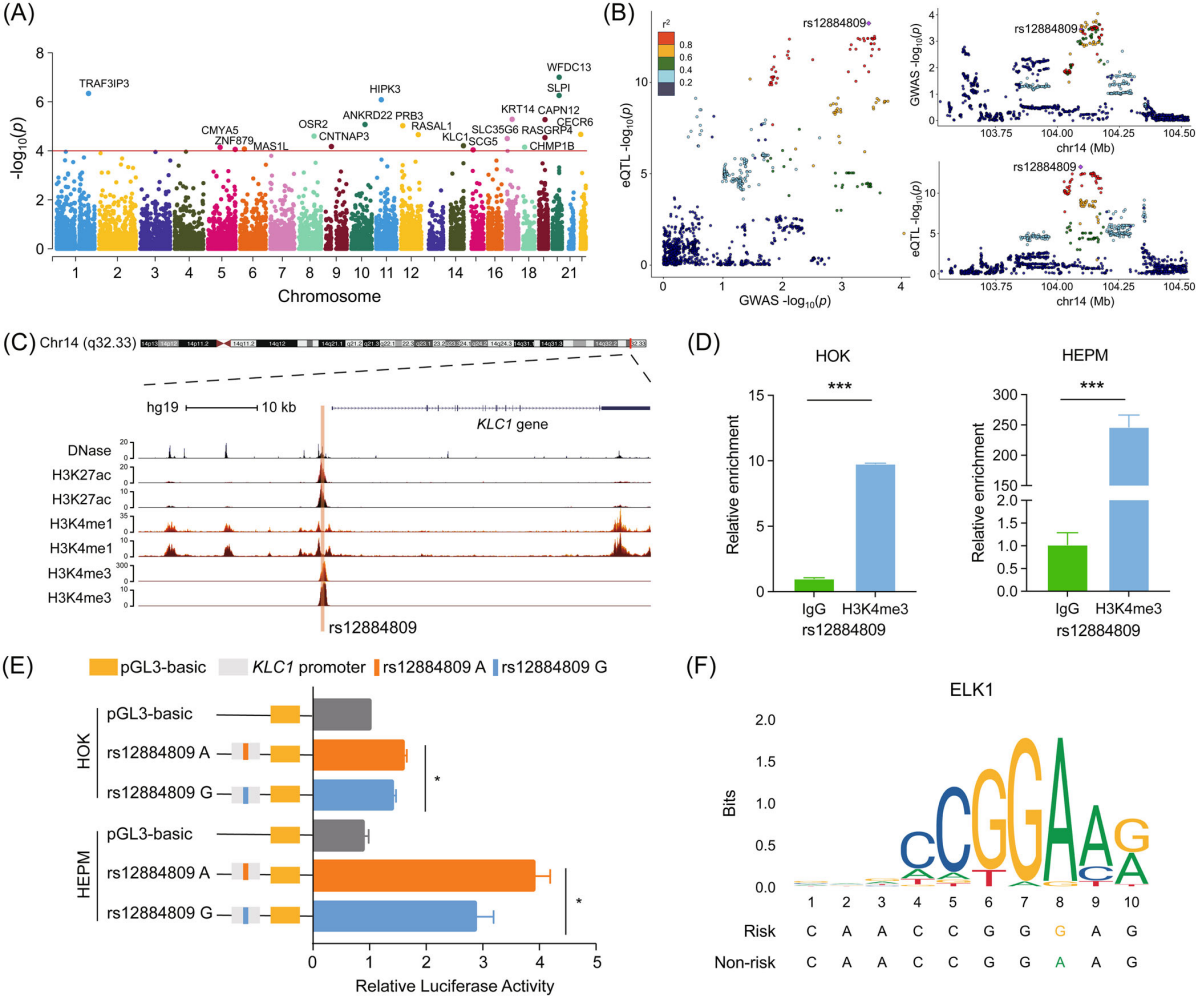
Genetic associations by both genome‐wide association studies (GWAS) and transcriptome‐wide association studies (TWAS) and allele‐specific effect of rs12884809. (A) Manhattan plot of the cross‐tissue TWAS results for NSCL/P. The red horizontal line indicates genes with nominal significance (*p* < 1 × 10^−4^). (B) GWAS and expression quantitative trait loci (eQTL) associations for *KLC1*. Left panel: The relationship between GWAS and eQTL is presented as a scatter plot, of which the x‐axis represents GWAS *p*‐value in −log_10_ scale for NSCL/P and the *y*‐axis represents the whole blood eQTL *p*‐value in −log_10_ scale. Right panel: The regional association plots present the GWAS association (upper panel) and the eQTL association (lower panel) for rs12884809 in whole blood. (C) Overview of the H3K4me1, H3K27ac, and H3K4me3 chromatin modifications, and DNase cluster distribution in the regions surrounding rs12884809 of the early human craniofacial tissues supported from the UCSC genome browser. Rs12884809 is indicated by the orange vertical line. (D) Histone H3K4me3 enrichment at SNP rs12884809 site in human oral keratinocyte (HOK) and human palatal mesenchymal (HEPM) cells by chromatin immunoprecipitation followed by quantitative PCR (ChIP‐qPCR) analysis. The *p* values were calculated using a two‐tailed unpaired Student's *t*‐test. *** indicates *p* < 0.001. (E) Dual‐luciferase reporter assay of promoter activity affected by rs12884809. Rs12884809 had allelic effects on promoter activity in HOK and HEPM cells, where A allele had higher activity than G allele. The *p* values were calculated using a two‐tailed unpaired Student's *t*‐test. * indicates *p* < 0.05. (F) The ELK1 binding site to the A allele of rs12884809 was predicted using PERFECTOS‐APE.

We conducted both cross‐ and single‐tissue TWAS to investigate the relationship between gene expression and NSCL/P. Through UTMOST analysis, 20 genes showed significant associations with NSCL/P (*p* < 1 × 10^−4^) across multiple tissues (Figure 1A, Table S1). To better characterize these genes, we assessed their tissue‐specific associations using FUSION and found six significant genes were significantly associated with NSCL/P risk in at least three tissues (*p* < 0.05, Tables S1 and S2). Notably, *TRAF3IP3* and *OSR2* have been previously reported to be associated with NSCL/P [7, 9, 10], while *WFDC13*, *SLPI*, *RASGRP4* and *KLC1* were newly identified. Current studies have indicated that *WFDC13*, a member of the telomeric cluster, showed significant variation in the testis and proximal epididymis during reproductive aging; *SLPI* is critical for tissue repair, including intra‐oral wound healing [11], *RasGRP4*, an activator of Ras protein, is involved in inflammation and immune activation [12]; *KLC1* is a key component of kinesin proteins, crucial for intracellular transport and highly enriched in brain and neural tissues [13]. Further studies would put more effort into exploring their roles in the occurrence and development of NSCL/P.

### Genetic regulation of rs12884809 on *KLC1*

Next, the colocalization analysis by combining GWAS and eQTL identified that *KLC1* at 14q32.3 shared the same genetic signals between NSCL/P GWAS and eQTL, with posterior probabilities PP4 > 0.75 (Table S3), but not observed in the other three genes. Notably, rs12884809 assigned to *KLC1* was significantly associated with NSCL/P risk (*OR* = 0.79, 95% CI: 0.69–0.90, *p* = 3.55 × 10^−4^), and *KLC1* expression in whole blood (*β* = 0.20, *p* = 5.77 × 10^−^^14^, Figure 1B, Table S3). Besides, SNPs in strong LD with rs12884809 also showed significant associations with NSCL/P risk and *KLC1* expression (Figure 1B). Novas et al. showed that *KLC1* regulated ciliary length, which is vital for embryonic development [14]. Malfunctions in cilia can lead to ciliopathies, including craniofacial abnormalities ranging from minor midline defects to severe clefts [15]. Additionally, the DECIPHER database identified four individuals with cleft palate and chromosomal abnormalities in this region [16]. Despite these associations, *KLC1*'s role in NSCL/P development remains unclear.

3DSNP and Haploreg showed that rs12884809 is located in chromatin regions exhibiting promoter and transcription regulatory activity (Table S4, Figure S2A), specifically within the promoter region of *KLC1*, marked by H3K4me3 in early human craniofacial tissues (Figure 1C). Chromatin immunoprecipitation (ChIP) results further confirmed H3K4me3 enrichment at rs12884809 in human oral keratinocyte (HOK) and human palatal mesenchymal (HEPM) cells (Figure 1D), indicating its promoter activity. Luciferase‐based promoter assays showed that the rs12884809 A allele significantly increased promoter activity compared to the G allele (Figure 1E). Electrophoretic mobility shift assays (EMSA) also demonstrated stronger binding of nuclear extracts to the A allele than the G allele (Figure S2B).

Next, we used Cistrome and PERFECTOS‐APE to elucidate the transcription factor binding affinity affected by rs12884809. We observed that rs12884809 lies within ELK1 binding motifs with obvious binding difference between the G and A alleles (Figure 1F, Table S5). Super‐shift EMSA assays further supported ELK1's binding preference for the A allele (Figure S2C). ELK1, a member of the Ets family of transcription factors and the ternary complex factor subfamily, plays an important role in transcription regulation. In this study, we observed that *ELK1* knockdown reduced *KLC1* expression, while overexpression increased it in HOK and HEPM cells (Figure S2D,E). A prior study demonstrated that the *TBX22*‐73G>A variant disrupts the Ets‐1 binding site, significantly reducing *TBX22* promoter activity and leading to cleft lip and palate defects [17]. These results suggested that the rs12884809 G>A variant altered promoter activity by affecting ELK1 binding, thereby regulating *KLC1* expression.

### *KLC1* deficiency leads to craniofacial abnormalities in zebrafish and disrupts cell proliferation, migration, and apoptosis

We initially observed continuous *klc1* expression during lip and palate development (E10.5 d to E15.5 d) and in craniofacial tissues (E10.5 d to E14.5 d) of the mouse model (Figure S3A,B); notably, *KLC1* was significantly downregulated in dental pulp stem cells derived from NSCL/P patients compared to controls (Figure S3C).

**FIGURE 2:**
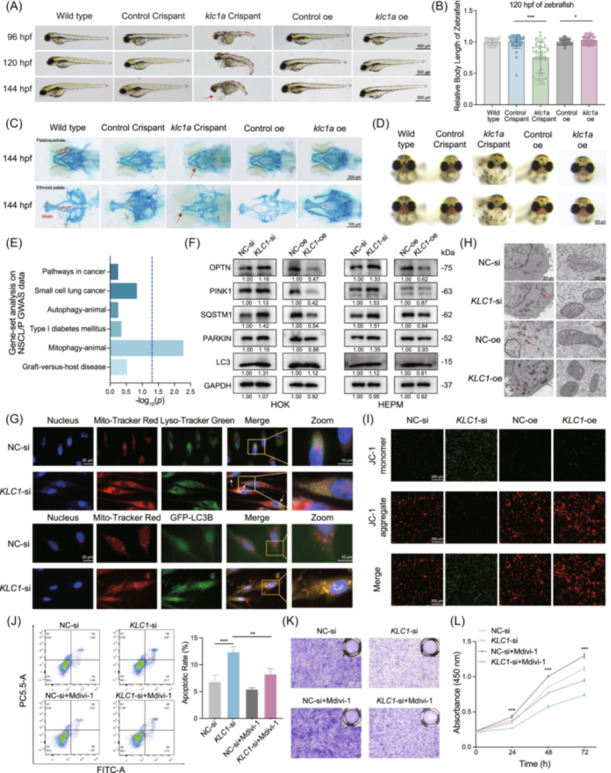
In vivo and in vitro role of KLC1 and its regulation of mitophagy. (A) The *klc1a* crispant embryos showed a shorter body length, curvature of the spine, craniomaxillofacial deficiency (indicated by red dashed lines) and edema around the heart (indicated by red arrow). (B) Quantitative analysis of body length in 120 hpf zebrafish embryos. (C) Upper panel: Alcian blue staining was used to assess the length of the palatoquadrate in zebrafish embryos at 144 hpf. The *klc1a* crispant embryos displayed a shorter palatoquadrate length compared to the other groups (indicated by red arrow). Lower panel: Alcian blue staining of the length and width of the ethmoid palate in zebrafish embryos at 144 hpf. The *klc1a* crispant embryos showed a cleft in the ethmoid palate (indicated by red arrow). (D) The lip morphology of zebrafish. The *klc1a* crispant embryos exhibited multiple lip deformities and clefts (indicated by red dashed lines), whereas zebrafish in other groups showed intact, oval‐shaped lips. oe, overexpression. (E) Gene‐set analysis on the NSCL/P GWAS data. Blue dotted lines represent statistically significant differences. (F) Western blot validation of mitophagy‐related genes in HOK and HEPM cells with *KLC1* knockdown (si) or overexpression (oe). Relative quantification numbers of protein expression are shown below the lanes. (G) Live cell imaging showed co‐localization of lysosomes and mitochondria (Lyso‐Tracker and Mito‐Tracker, indicated by white arrows), and autophagosomes and mitochondria (GFP‐LC3B and Mito‐Tracter, indicated by red arrows) in HEPM cells. (H) Transmission electron microscopy (TEM) images showed mitophagy changes in HEPM cells with or without *KLC1* knockdown/overexpression (scale bar = 2 μm and 500 nm). Red arrows indicate autophagosomes involved in the mitophagy process. (I) JC‐1 staining demonstrated changes in mitochondrial membrane potential in HEPM cells. *KLC1* knockdown led to (J) increased apoptosis, (K) reduced migration, and (L) proliferation in HEPM cells, all of which were partially rescued by treatment with the mitophagy inhibitor Mdivi‐1. ns, not significant, * indicates *p* < 0.05, ** indicates *p* < 0.01, *** indicates *p* < 0.001.

To explore *KLC1*'s biological role, we generated CRISPR/Cas9‐based targeted *klc1a* knockdown zebrafish models and *klc1a* overexpression models (Figure S4A). The *klc1a* knockdown embryos exhibited lower survival rates at 48 hours post‐fertilization (hpf), reduced hatching rates at 72 hpf, and increased abnormalities at 96 hpf (Figure S4B−D). These embryos also showed spinal curvature, craniofacial deficiencies, heart edema, and shorter body length compared to controls, while overexpression models displayed increased body length without deformities (Figure 2A,B). Iridophore distribution was also reduced in the knockdown group (Figure S4E). In zebrafish, the palatoquadrate and ethmoid plate correspond to the human maxilla and palate, both of which are relevant to NSCL/P [18]. *Klc1a* knockdown embryos showed reduced palatoquadrate length and smaller ethmoid plate dimensions, whereas *klc1a* overexpression increased these measurements (Figure 2C). Knockdown embryos also exhibited multiple lip deformities (Figure 2D).

In cell models, *KLC1* knockdown inhibited proliferation, migration, increased apoptosis in HOK and HEPM cells, whereas overexpression enhanced proliferation, migration, and reduced apoptosis (Figure S5). These findings suggest that *KLC1* is crucial for craniomaxillofacial development.

### *KLC1* regulating mitophagy in NSCL/P development

To further elucidate the mechanisms through which the *KLC1* influences NSCL/P, we performed RNA‐seq on *KLC1* knockdown cell models. In HOK and HEPM cells, *KLC1* knockdown significantly dysregulated 157 and 699 genes, respectively (Figure S6A,B). KEGG analysis identified six significant pathways, including autophagy and mitophagy, with false discovery rate (FDR) below 0.05 (Figure S6C,D). Further, the gene set enrichment analysis using GWAS data revealed only the mitophagy pathway as statistically significant (Figure 2E). Especially, gene set variation analysis (GSVA) analysis showed upregulated mitophagy scores in *KLC1*‐knockdown cells (Figure S6E), and *KLC1* expression was negatively correlated with mitophagy scores (Figure S6F). Moreover, we observed that mitophagy hub genes were upregulated in response to *KLC1* knockdown (Figures 2F, S7). Mitophagy assays indicated increased co‐localization of lysosomes with mitochondria and GFP‐LC3 with mitochondria in *KLC1*‐knockdown cells (Figures 2G, S8A). In addition, both transmission electron microscopy (Figures 2H, S8B) and JC‐1 staining analysis (Figures 2I, S8C) confirmed increased mitophagy with *KLC1* knockdown and decreased with overexpression. Mdivi‐1, a known mitophagy inhibitor [19], was further used to investigate the role of *KLC1* on mediating mitophagy. Treatment with Mdivi‐1 alongside *KLC1* siRNAs partially rescued the impact of *KLC1* knockdown on apoptosis, proliferation, and migration (Figures 2J−L, S9).

## CONCLUSION

In summary, this study utilized the TWAS approach to integrate eQTL data with NSCL/P GWAS data, identifying key loci and genes involved in NSCL/P development. Through in vivo and in vitro experiments, we demonstrated rs12884809 G>A enhanced the binding of the transcription factor ELK1 to the *KLC1* promoter region, which increased *KLC1* expression. On the other hand, reduced *KLC1* expression promoted mitophagy, which could lead to decreased cell proliferation and migration, increased apoptosis, and consequently a higher risk of NSCL/P. This study provides valuable insights for both the fundamental understanding and clinical prevention of NSCL/P.

## AUTHOR CONTRIBUTIONS

**Shu Lou**: Conceptualization; methodology; writing—review and editing; writing—original draft. **Guirong Zhu**: Writing—original draft; conceptualization; methodology. **Changyue Xing**: Validation; data curation. **Shushu Hao**: Visualization; validation. **Junyan Lin**: Validation. **Jiayi Xu**: Validation. **Dandan Li**: Data curation. **Yifei Du**: Data curation. **Congbo Mi**: Resources. **Lian Sun**: Resources. **Lin Wang**: Investigation. **Meilin Wang**: Investigation. **Mulong Du**: Investigation. **Yongchu Pan**: Investigation; funding acquisition; supervision.

## CONFLICT OF INTEREST STATEMENT

The authors declare no conflicts of interest.

## ETHICS STATEMENT

This study was approved by the Institutional Review Board of Nanjing Medical University (NJMUERC [2008] no. 20) and signed informed consents were obtained from participants or their legal guardians.

## Supporting information


**Figure S1.** Quantile‐quantile plot and Manhattan plot of the GWAS results.
**Figure S2.** The function of rs12884809 and transcription factor.
**Figure S3.** *KLC1* expression pattern in NSCL/P‐related tissues.
**Figure S4.** Perturbation of *klc1a* in developing zebrafish embryos.
**Figure S5.** Effect of *KLC1* on celluar behaviors.
**Figure S6.** Pathway enrichment for the genes regulated by *KLC1*.
**Figure S7.** qPCR validation of mitophagy‐related genes in HOK and HEPM cells.
**Figure S8.** Regulation of Mitophagy by *KLC1*.
**Figure S9.** Impact of *KLC1* on cellular behaviors via mitophagy regulation involving NSCL/P.

**Table S1.** Significant genes in the TWAS analysis.
**Table S2.** The significant results of FUSION for candidate susceptibility genes.
**Table S3.** Results of colocalization analysis for the significant genes.
**Table S4.** Comprehensive annotation of rs12884809.
**Table S5.** The transcription factor binding of rs12884809 predicted by PERFECTOS‐APE.
**Table S6.** Basic information of participants in each data set.

## Data Availability

The data that support the findings of this study are available from the corresponding author upon reasonable request. All the sequencing data have been deposited in NCBI and the Genome Variation Map under submission number PRJNA1184036 and GVM000904 (https://www.ncbi.nlm.nih.gov/sra/?term=PRJNA1184036; https://ngdc.cncb.ac.cn/gvm/getProjectDetail?project=GVM000904). Supplementary materials (methods, figures, tables, graphical abstract, slides, videos, Chinese translated version and update materials) may be found in the online DOI or iMeta Science http://www.imeta.science/.
