# Supplementary material for: Transcriptome‐wide association identifies KLC1 as a regulator of mitophagy in non‐syndromic cleft lip with or without palate
Source: Imeta. 2024 Dec 20;3(6):e262. doi: 10.1002/imt2.262 (PMC11683466; doi:10.1002/imt2.262)
Supplement: Supplementary file 1 — Figure S1. Quantile‐quantile plot and Manhattan plot of the GWAS results. Figure S2. The function of rs12884809 and transcription factor. Figure S3. KLC1 expression pattern in NSCL/P‐related tissues. Figure S4. Perturbation of klc1a in developing zebrafish embryos. Figure S5. Effect of KLC1 on celluar behaviors. Figure S6. Pathway enrichment for the genes regulated by KLC1. Figure S7. qPCR validation of mitophagy‐related genes in HOK and HEPM cells. Figure S8. Regulation of Mitophagy by KLC1. Figure S9. Impact of KLC1 on cellular behaviors via mitophagy regulation involving NSCL/P. [file IMT2-3-e262-s002.docx]

Supporting information to

Transcriptome-Wide Association Identifies *KLC1* as a Regulator of Mitophagy in Non-Syndromic Cleft Lip with or without Palate

Running title: *KLC1* Modulates Mitophagy in NSCL/P

Shu Lou^1,2,3#^, Guirong Zhu^1,3#^, Changyue Xing^1,3^, Shushu Hao^1,3^, Junyan Lin^1,3^, Jiayi Xu^1,3^, Dandan Li^1,2,3^, Yifei Du^1,3^, Congbo Mi^4^, Lian Sun^1,2,3^, Lin Wang^1,2,3,5^, Meilin Wang^1,5,6*^, Mulong Du^6,7*^, Yongchu Pan^1,2,3,5*^

^1^State Key Laboratory of Cultivation Base of Research, Prevention and Treatment for Oral Diseases, Nanjing Medical University, Nanjing, 210029, China

^2^Department of Orthodontics, Affiliated Hospital of Stomatology, Nanjing Medical University, Nanjing, 210029, China

^3^Jiangsu Province Engineering Research Center of Stomatological Translational Medicine, Nanjing Medical University, Nanjing, 210029, China

^4^The First Affiliated Hospital of Xinjiang Medical University, Wulumuqi, 830054, China

^5^State Key Laboratory of Reproductive Medicine, Nanjing Medical University, Nanjing, 211166, China

^6^Department of Genetic Toxicology, the Key Laboratory of Modern Toxicology of Ministry of Education, Center for Global Health, School of Public Health, Nanjing Medical University, Nanjing, 211166, China.

^7^Department of Biostatistics, Center for Global Health, School of Public Health, Nanjing Medical University, Nanjing, 211166, China.

^#^These authors contributed equally: Shu Lou, Guirong Zhu

*Correspondence: panyongchu@njmu.edu.cn (Yongchu Pan), drdumulong@njmu.edu.cn (Mulong Du), and mwang@njmu.edu.cn (Meilin Wang)

Materials and Methods

Study population and GWAS summary data

The NSCL/P GWAS study comprised 1,069 cases and 1,724 controls (Table S6), including 504 cases and 455 controls (Stage I) using Affymetrix Axiom Genome-Wide CHB1 and CHB2 arrays, and 565 cases and 1,269 controls (Stage II) using Illumina Infinium Asian Screening Array. Comprehensive details of quality control analysis have been described before [1, 2]. The genotype data were further imputed with IMPUTE2 and phased with SHAPEIT. A total of 1000 Genomes Project reference panel (the Phase I integrated variant set release, v3, across 286 Asians) was set as the reference. Additional inclusion criteria included the following SNPs with high imputation quality (INFO) ≥ 0.8; minor allele frequency (MAF) ≥ 0.05; Hardy-Weinberg disequilibrium *p* ≥ 1 × 10^−4^ and included in the HapMap Phase 2 version. This study was approved by the Institutional Review Board of Nanjing Medical University (NJMUERC [2008] no. 20) and signed informed consents were obtained from participants or their legal guardians.

To explore the genetic associations in a multiethnic population, we obtained the NSCL/P GWAS summary statistics from the FinnGen database (https://www.finngen.fi/en), which are limited to individuals of European descent. The study included 400 NSCL/P cases and 342,099 controls. The SNP locations are based on the Genome Reference Consortium Human Build 37.

Cross-tissue TWAS analysis using UTMOST

Cross-tissue TWAS was performed through the utilization of UTMOST, which incorporated genotype and normalized gene expression data from 450 individuals in the GTEx V7. First, pre-determined covariance matrices were obtained from UTMOST website (https://github.com/Joker-Jerome/UTMOST). Then, using NSCL/P GWAS summary data and genetic-expression weights, single-tissue association studies for 44 tissues were carried out. Finally, a joint GBJ test was used to integrate gene-trait relationships in 44 tissues. The transcriptome-wide significance of the joint test was determined to be *p*-value < 1 × 10^-4^.

Single tissue TWAS analysis using FUSION

The single tissue TWAS analysis was conducted using FUSION to reduce false-positive errors. On the official FUSION website (http://gusevlab.org/projects/fusion/), 44 pre-computed predictive models for GTEx V7 tissues were downloaded. Then, we evaluated the association between each gene and NSCL/P by combining the previously computed weights for gene expression with the NSCL/P GWAS summary data. The LD references were acquired from a diverse culture of Europeans and East Asians in the 1000 Genome Project. Finally, we chose the significant UTMOST genes with *p*_FUSION_ less than 0.05 as the most deserving of future study.

Colocalization of eQTL and GWAS signals

To identify trait-associated SNPs in genes, a Bayesian colocalization was executed by the R package ‘coloc’. The results of this procedure included five posterior probabilities (PP0, no causal variant; PP1, causal variant for GWAS only; PP2, causal variant for eQTL only; PP3, two distinct causal variants; PP4, only one common causal variant) for each hypothesis. A greater PP4 score suggests a greater likelihood of a shared causal signal for both GWAS and eQTL. SNPs were considered to be colocalized for eQTL and NSCL/P if the PP4 was greater than or equal to 0.75. We performed the colocalization in the tissue with the most significant *p*_FUSION_ for each identified gene. Scatterplot plots were generated with LocusCompare (http://locuscompare.com/) [3].

In silico annotations for SNPs and candidate genes

HaploReg v4.1 (https://pubs.broadinstitute.org/mammals/haploreg/haploreg.php), RegulomeDB (http://www.regulomedb.org/), and GWAVA (https://www.sanger.ac.uk/sanger/StatGen_Gwava/) databases were used to annotate the functional impact of SNPs. HaploReg v4.1 provides information on all sites with a linkage disequilibrium greater than or equal to 0.8, including histone marks such as H3k4me1, H3k4me3, and H3k27ac, as well as eQTL information. RegulomeDB can be used to predict DNA features and regulatory elements. GWAVA aims to predict the functional impact of non-coding genetic variants based on the comprehensive annotation of non-coding elements and whole-genome properties. The UCSC Genome Browser (http://genome.ucsc.edu/) was used to visualize and compare the ChIP-seq tracks of human embryonic craniofacial tissues (GSE97752) for SNPs. 3D chromatin looping data (http://cbportal.org/3dsnp/) was applied to correspond the promising SNPs to their chromatin state. Genotype-gene expression was referred from GTEx (https://www.gtexportal.org/home/index.html).

Transcription factor binding site analysis was executed with the Opera House (https://opera.autosome.org/perfectosape/description). The ChIP-seq data (CistromeDB: 45636) of the candidate transcription factors were analyzed using the Cistrome Data Browser (Cistrome DB, http://cistrome.org/db/#/). The visualization of the ChIP-seq data was presented using the genome browser WashU Epigenome Browser (http://epigenomegateway.wustl.edu/).

We also queried the GSE67985 database, which contained RNA-Seq data on craniofacial structures during mouse embryo development, to identify the expression patterns of candidate genes. Additionally, differential expression analysis was performed based on the GSE42589 database with dental pulp stem cells of seven NSCL/P cases and six controls.

Cell culture

Human palatal mesenchymal cells (HEPM) were cultured in Minimum Essential Medium α (MEMα, Gibco, USA) supplemented with 10% fetal bovine serum (FBS, Gibco, USA) and 1% penicillin/streptomycin (Gibco, USA) in 5% CO_2_ at 37°C. Human oral keratinocyte (HOK) cells were cultured in Dulbecco’s modified Eagle’s medium (DMEM, Gibco, USA) with 10% FBS and 1% penicillin-streptomycin at 37°C in 5% CO_2_.

Plasmid constructions construct and transfection

Rs12884809 A or G allele in the *KLC1* promoters (-2000 to +1 to the transcription start site) was designed and cloned into the pGL3-Basic luciferase vector to evaluate if rs12884809 could affect the promoter activity (Promega, USA). The small interference RNA (siRNA) oligonucleotides targeting *KLC1* were synthesized and purchased (GenePharma, China). The siRNA sequences used were as follows: ELK1-homo-1396 (5'-CUUCCAGUUUCCAUCCAGUTT-3', 5'-ACUGGAUGGAAACUGGAAGTT-3'), ELK1-homo-306 (5'-GUGGUGAAUUCAAGCUGGUTT-3', 5'-ACCAGCUUGAAUUCACCACTT-3'), ELK1-homo-901 (5'-GAAAUCGGAAGAGCUUAAUTT-3', 5'-AUUAAGCUCUUCCGAUUUCTT-3'), KLC1-homo-1339 (5'-GGGCAAGUAUGAAGAAGUATT-3', 5'-UACUUCUUCAUACUUGCCCTT-3'), KLC1-homo-385 (5'-GCUGGAAGCUUUGAAGAAUTT-3', 5'-UAUUCUUCAAAGCUUCCAGCTT-3'), KLC1-homo-1571 (5'- GCUGAAGAAAGAGAAGAAUTT-3', 5'-AUUCUUCUCUUUCUUCAGCTT-3'), Negative Control (5'-UUCUCCGAACGUGUCACGUTT-3', 5'-ACGUGACACGUUCGGAGAATT-3'). The *KLC1* overexpression and control vector were obtained from Promega (USA). All transfections into HEPM and HOK were performed using Lipofectamine 2000 (Invitrogen, USA) according to the manufacturer's instructions.

Luciferase activity assay

The luciferase activity was quantified 48 h after transfection with Dual-Luciferase Reporter Assay Kit (Vazyme, China). The ratio of firefly luciferase to *Renilla* luciferase activity was considered a relative reporter activity. The experiments were performed in triplicate.

RNA extraction and quantitative Real-Time PCR

The total RNA was isolated using TRIzol reagent (Vazyme, China) according to the manufacturer’s protocol. Complementary deoxyribonucleic acid (cDNA) was synthesized using 1 μg RNA using the HiScript® Q RT SuperMix for qPCR (Vazyme, China). Then, qPCR reactions were performed using the SYBR Green master mix (Vazyme, China) on the Quantstudio 7 Flex Real-Time PCR System (Applied Biosystems, CA). All reactions were conducted in triplicate, and the data were analyzed by the 2^-ΔΔCt^ method.

Chromatin immunoprecipitation assay

Cells were crosslinked with 1% formaldehyde and quenched in a glycine solution. ChIP assay was performed by Magna ChIP™ A/G Chromatin Immunoprecipitation Kit (Millipore, USA), according to the manufacturer’s instructions. Anti-H3K4me3 antibody (Abcam, USA) and normal mouse IgG (Millipore, USA) were used for immunoprecipitation. ChIP-enriched DNA samples were quantified by qPCR to determine the H3K4me3 binding sites of rs12884809. The value was shown as relative enrichment normalized to IgG. Primers used for ChIP-qPCR were as follows:

rs12884809-F: TGGAAGGGAAGCTGCCGAAG, rs12884809-R: ATCAGGTGGGCGGTCACAG.

Electrophoretic mobility shift assay (EMSA)

Complementary DNA oligonucleotides (23 bp) centered on the predicted binding peak initiation sequence of rs12884809 were synthesized (Bosteed, China) and labelled with biotin on their 3′ ends: sense 5′GTTACAACCGG[A/G]AGGCCCGCTGG 3′-biotin, anti- sense 5′ CCAGCGGGCCT[T/C]CCGGTTGTAAC 3′-biotin. Non-biotin-labelled probes containing the same sequences for competitive inhibition experiments. Cell nuclear proteins were obtained using a Nuclear and Cytoplasmic Protein Extraction Kit (KeyGEN Biotech, China). EMSA was performed using LightShift Chemiluminescent EMSA Kit (ThermoFisher, USA). Unlabeled probes were added to the binding reaction for 20 min at 50-fold excess over the labelled probe prior to the addition of the labelled probe. We observe changes in the electrophoretic mobility rates in 6.5% polyacrylamide gels. In addition, to detect particular DNA-protein products, we used antibodies against ELK1 (ab125085, Abcam, USA) by super EMSA.

Cell proliferation assays

The transfected HOK and HEPM were seeded in 96‐well plates with a 100 μl culture medium to assess proliferation. After transfecting for 0, 24, 48, and 72 h, 10 μl of Cell Counting Kit‐8 (CCK-8, NCM, China) were added to each well, and cells were then cultured at 37°C in 5% CO_2_ for 2 h. Absorbance was read at 450 nm using SpectraMax 190 (MDC, USA). Three independent experiments were performed with technical triplicates.

Cell apoptosis assays

To measure apoptosis, the cells were collected and stained with Annexin V-FITC/Propidium Iodide Kit (KeyGEN Biotech, China) after transfecting for 48 h. The cells were incubated in the dark at 20-25°C for 10 min. FlowJo™ v10 software (FlowJo LLC, USA) was applied for the flow cytometric analysis. Each experiment had three technical replicates.

Cell migration assays

For the migration assays, 1 × 10^5^ cells in serum-free media were plated into the upper chamber of an insert after transfection. The bottom wells were filled with a 700 μl medium containing 20% FBS. After incubation for 24 h, cells that had migrated through the membrane were fixed with 4% paraformaldehyde, stained with crystal violet solution, and then imaged and counted. All the experiments were independently repeated three times.

Zebrafish studies

Zebrafish were raised on a 14/10 h light/dark cycle at 28.5 °C in the zebrafish facility of the Model Animal Research Center, Nanjing Medical University. The zebrafish *klc1a* gene sequences were obtained from the zebrafish information network (www.zfin.org). Single guide RNAs (sgRNAs) were designed using the CRISPRscan algorithm 22 and synthesized by Hunter Biotech (Hunter Biotech, China). For the CRISPR/Cas9 microinjection, Cas9 protein (GenScript, Z03388-100) and sgRNA mix were prepared and zebrafish embryos were injected directly with 800 ng/μl of Cas9 and 320 ng/μl of gRNA per embryo, respectively. A non-specific gRNA was used to generate control-injected zebrafish. To confirm genome editing, sanger sequencing was applied. We chose the feasibility of CRISPR/Cas9-based F0 (crispant) for functional validation of *klc1a*. The coding sequence (CDS) of zebrafish *klc1a* was amplified and cloned into pcDNA™3.1(+). All constructs were confirmed by Sanger sequencing. For the over-expression experiment, 100 ng/µL 1 nl zebrafish *klc1a* mRNA per embryo was injected into one-cell stage embryos.

Zebrafish were imaged with transmitted light at different points in time. Zebrafish embryos were fixed in ethanol, and then stained with Alcian blue. Zebrafish were bleached to remove pigment by incubation in 3% hydrogen peroxide (H_2_O_2_) and 2% potassium hydroxide (KOH). Specimens were stored and analyzed in 100% glycerol. The length and amount were measured with ImageJ software (NIH, USA).

RNA-seq for mouse embryonic and *KLC1*-knockdown cells

We extracted total RNA with TRIzol reagent (Invitrogen, USA) from mice lip and palate from E10.5d to E15.5d, HOK and HEPM with *KLC1*-knockdown and control cells and carried out quality control. The Hiseq3000 sequencing platform (Illumina, CA, USA) in paired-end mode was used for mRNA-seq in 10G depth. After trimming out joint sequences and low-quality segments, the sequences were mapped to the mouse genome (mm10) or human genome (hg19). Finally, we assigned the Fragments Per Kilobase of exon per Million fragments mapped (FPKM) as the mRNA expression level. A co-expression analysis of transcription factors and candidate genes was performed, where the correlation coefficient was determined using Pearson analysis, and the data was visualized through GraphPad Prism7 software (GraphPad Software, CA, USA).

Pathway enrichment analysis

For RNA-seq results of HOK and HEPM knockdown, FDR < 0.05 and |log_2_FC| > 1 were used to identify genes for downstream analysis. The Kyoto Encyclopedia of Genes and Genomes (KEGG) pathways analysis was performed by the “clusterProfiler” package in R to explore the biological functional roles of differentially expressed genes in 2 cell lines. We also used the “GSVA” R package to perform gene set variation analysis (GSVA) enrichment analysis to determine differences in biological processes in the Reactome database [4]. GSVA could yield single sample enrichment scores. To further investigate their association with NSCL/P, gene-set analysis on the NSCL/P GWAS data was conducted using MAGMA software.

Western blots

The radio immunoprecipitation assay (RIPA) lysis buffer were added to each cell fraction with mixture of protease inhibitor cocktail. The concentration of proteins in different sample was measured by BCA (bicinchoninic acid) assay kit method. Total protein was separated on a 10% SDS‐PAGE, and then the gel was transferred to polyvinylidene difluoride membranes. Afterwards, the membrane was blocked by skim milk which diluted by TBST. Then, membranes were incubated with primary antibodies of optineurin (OPTN, 1:1000, ab213556, Abcam), sequestosome‐1 (SQSTM1/p62, 1:1000, ab207305, Abcam), Parkin (1:2000,14060-1-AP, Proteintech), Pink1 (1:1000, ab216144, abcam) , LC3B (1:1000, 14600-1-AP, Proteintech), GAPDH (1:5000,10494-1-AP, Proteintech), at 4°C overnight. Non‐horseradish peroxidase (HRP) conjugated primary antibody was incubated with HRP‐conjugated secondary antibody and the protein‐antibody binding reaction in membrane was detected by the enhanced chemiluminescence assays.

Transmission electron microscopy (TEM)

To prepare the TEM samples, cell clusters were collected and fixed in electron microscope fixative for 2-4 h. Cells were then centrifuged, supernatants discarded and added to 0.1 M phosphate buffer PB (PH 7.4), mixed and rinsed, and then preembedded in agar. 1% osmium acid was fixed for 2 h at room temperature away from the light, followed by alcohol dehydration at room temperature in a gradient, and acetone infiltration embedding, and then solidified for 48 h in an oven at 60 °C. The slices were sectioned and retrieved with a copper mesh, and stained with 1% uranyl acetate saturated alcohol and 2% lead citrate solution away from carbon dioxide. The sections were stained with 1% uranyl acetate saturated alcohol solution and 2% lead citrate solution to avoid carbon dioxide staining. Mitochondrial images of the cells were obtained using a transmission electron microscope system (HT7800, HITACHI).

Mitochondrial membrane potential (MMP) measurement

Mitochondrial membrane potential was measured using the JC-1 staining kit. JC-1 polymer (excitation wavelength: 585 nm; emission wavelength: 590 nm) emits red fluorescence, which indicates a high mitochondrial membrane potential. JC-1 monomer (excitation wavelength: 514 nm; emission wavelength: 529 nm) emits green fluorescence, which indicates a low mitochondrial membrane potential. Changes of MMP were assessed by the ratio of fluorescence intensity between green colour and red colour. MMP in cultured cells was measured by JC-1 Assay Kit (C2003S, Beyotime, China). Cultured cells were incubated with JC-1 stain for 30 min. The fluorescence intensity of JC-1 stain was then recorded using a confocal microscope (Zeiss, Germany). Fluorescence images were captured by a fluorescence microscope (Zeiss).

Mitophagy assay

To detect the level of mitophagy, the colocalization of mitochondria with autophagosome was achieved by RFP-Mito (C1035, Beyotime, China) and GFP-LC3 (C3006, Beyotime, China) lentivirus transfection, and mitochondria with lysosome was achieved by RFP-Mito and Lyso-Tracker (L7526, Thermo, USA), respectively. Live cell nuclear was stained by Hoechst 33342 (C1028, Beyotime, China).

Statistical analysis

SNPTEST 2.5.6, PLINK 1.09 and R 3.6.2 (http://www. r-project.org/) were used for statistical analysis. Genetic association tests were performed using additive logistic regression models in the software SNPTEST v2.5.6. We then performed a fixed-effects inverse-variance-weighted meta-analysis of the summary statistics from both stages using METAL. A Manhattan plot of –log_10_*p* was generated using the ggplot2 package in the R software. For all graphs, statistical analyses were performed using a two-tailed unpaired Student’s *t*-test. Before *t*-test, the normal distribution of all data was checked using a normality test, and the equality of variances was checked using *F*-test. Data were considered statistically significant at *p* < 0.05.

References:

1. Sun, Yimin, Yongqing Huang, Aihua Yin, Yongchu Pan, Yirui Wang, Cheng Wang, Yong Du, et al. 2015. “Genome-wide association study identifies a new susceptibility locus for cleft lip with or without a cleft palate.” *Nature Communications* 6: 6414. <https://doi.org/10.1038/ncomms7414>

2. Lou, Shu, Jing Yang, Guirong Zhu, Dandan Li, Lan Ma, Lin Wang, Yongchu Pan. 2024. “Integrative Multi-omics Analysis Identifies Genetic Variants Contributing to Non-syndromic Cleft Lip with or without Cleft Palate.” *Chin J Dent Res* 27: 65-73. <https://doi.org/10.3290/j.cjdr.b5136745>

3. Liu, Boxiang, Michael J. Gloudemans, Abhiram S. Rao, Erik Ingelsson, Stephen B. Montgomery. 2019. “Abundant associations with gene expression complicate GWAS follow-up.” *Nature Genetics* 51: 768-769. <https://doi.org/10.1038/s41588-019-0404-0>

4. Hänzelmann, Sonja, Robert Castelo, Justin Guinney. 2013. “GSVA: gene set variation analysis for microarray and RNA-Seq data.” *BMC Bioinformatics* 14: 7. <https://doi.org/10.1186/1471-2105-14-7>

Figure S1. Quantile-quantile plot and manhattan plot of the GWAS results. (A) Quantile-quantile plot of Stage I, λ = 1.028. (B) Quantile-quantile plot of Stage II, λ = 1.020. (C) Quantile-quantile plot of Meta results, λ = 1.043. (D) Manhattan plot of Stage I. (E) Manhattan plot of Stage II. (F) Manhattan plot of GWAS meta-analysis results for NSCL/P. The red and blue horizontal lines indicate the level of genome-wide significance (*p* = 5 × 10^-8^) and nominal significance (*p* = 1 × 10^-5^).

Figure S2. The function of rs12884809 and transcription factor. (A) Rs12884809 was located in the promoter state and the corresponding score is 1.00 according to 3DSNP. (B) In electrophoretic mobility shift assays (EMSA), rs12884809[A] showed a higher binding affinity for transcription binding factors than the G allele in HOK cells and HEPM cells. The arrow indicated a DNA-protein complex. (C) *KLC1* was co-expressed with *ELK1* in HOK cells after knockdown and overexpression. (D) *KLC1* was co-expressed with *ELK1* in HEPM cells after knockdown and overexpression. (E) Supershift assay of transcription factor binding. The results indicated stronger ELK1 binding to the A allele in HOK and HEPM cells.**p* < 0.05, ***p* < 0.01, ****p* < 0.001

Figure S3. *KLC1* expression pattern in NSCL/P-related tissues. (A) *Klc1* expression in the lip and palate during mouse embryonic E10.5-E14.5 period by in-house RNA-seq. (B) Expression levels of *Klc1* in mouse craniofacial tissues from E10.5d to E14.5d according to GSE67985. Man.Distal: Mandibular distal location; Man.Proximal: Mandibular proximal location; Max.Distal: Maxillary distal location; Max. Proximal: Maxillary proximal location. (C) Differential *KLC1* expression of dental pulp stem cells in NSCL/P cases and controls in GSE42589.

Figure S4. Perturbation of *klc1a* in developing zebrafish embryos. (A) The knockdown and overexpression efficiency of *klc1a* in zebrafish. (B, C, D) The survival rate (B), hatching rate (C) and abnormal rate (D) of crispant and overexpression zebrafish at 48 hpf, 72 hpf and 96 hpf. (E) The distribution of iridophores in 120 hpf zebrafish embryos. OE, overexpression.

Figure S5. Effect of *KLC1* on celluar behaviors. (A, B) qRT-PCR was performed to detect the *KLC1* expression after knockdown and overexpression of *KLC1* in HOK and HEPM cells. (C, D) Cell counting kit-8 assay showed knockdown of *KLC1* inhibited the proliferation of HOK cells, while overexpression of *KLC1* promoted their proliferation. (E, F) Flow cytometric analysis demonstrated that knockdown of *KLC1* increased the apoptosis of HOK cells, and overexpression of *KLC1* decreased the apoptosis of HOK cells. (G, H) Knockdown of *KLC1* inhibited the migration of HOK cells by transwell assays. Meanwhile, overexpression of *KLC1* promoted the migration of HOK cells. (I, J) Cell counting kit-8 assay showed knockdown of *KLC1* inhibited the proliferation of HEPM cells, while overexpression of *KLC1* promoted their proliferation. (K, L) Flow cytometric analysis demonstrated that knockdown of *KLC1* increased the apoptosis of HEPM cells, and overexpression of *KLC1* decreased the apoptosis of HEPM cells. (M, N) Knockdown of *KLC1* inhibited the migration of HEPM cells by transwell assays. Meanwhile, overexpression of *KLC1* promoted the migration of HEPM cells. OE, overexpression. **p* < 0.05, ***p* < 0.01, ****p* < 0.001.

Figure S6. Pathway enrichment for the genes regulated by *KLC1*. (A) Heatmap of differently expressed genes between *KLC1*-knockdown and control HOK and HEPM cells. (B) Volcano plots of all genes. (C) The top 10 pathways in KEGG pathway enrichment for differentially expressed genes in HOK and HEPM cells. (D) List of differentially expressed genes enriched in KEGG pathways. KEGG, the Kyoto Encyclopedia of Genes and Genomes. ID, the pathway number in KEGG database. Sample number, the number of differentially expressed genes in this pathway. Background number, the number of annotated genes in this pathway. FDR, false discovery rate. (E) GSVA analyses of mitophagy pathway between *KLC1*-knockdown and control in HOK and HEPM cells. (F) Correlation between GSVA single sample enrichment score and *KLC1* expression level from RNA-seq in HOK and HEPM cells.

Figure S7. qPCR validation of mitophagy-related genes in HOK and HEPM cells. ns, not significant, **p* < 0.05, ***p* < 0.01, ****p* < 0.001, *****p* < 0.0001.

Figure S8. Regulation of Mitophagy by *KLC1*. (A) Live cell imaging showed co-localization of lysosomes and mitochondria (Lyso-Tracker and RFP-Mito) and autophagosomes and mitochondria (GFP-LC3 and RFP-Mito) in HOK cells. (B) TEM images showed mitophagy changes before and after KLC1 knockdown/overexpression (scale bar = 2 μm and 500 nm) in HOK cells. (C) JC‐1 staining demonstrated changes in mitochondrial membrane potential in HOK cells.

Figure S9. Impact of *KLC1* on cellular behaviors via mitophagy regulation involving NSCL/P. *KLC1* knockdown led to (A) increased apoptosis, (B) reduced migration, and (C) proliferation in HOK cells, all of which were partially rescued by treatment with the mitophagy inhibitor Mdivi-1. ns, not significant, ****p* < 0.001, *****p* < 0.0001.
